# Qualification and Registration

**Published:** 1912-09-07

**Authors:** 


### THE MEDICAL CURRICULUM.

### QUALIFICATION AND REGISTRATION.

The first thing a medical student has to do is to register himself as such, because the five years of study which are necessary in order to obtain a qualification are dated from the time of registration. For this purpose application must be made to the Registrar of the General Medical Council, 299 Oxford Street, London, W.

The student, however, cannot be registered until he has (1) passed a preliminary examination in Arts which is recognised by the General Medical Council, and (2) has commenced medical study.

A complete list of the recognised preliminary examinations may be obtained on application to the Registrar of the General Medical Council; and it is essential in any case to discover at a sufficiently early period what preliminary examinations are recognised by the particular examining body that it is proposed to work under. For example, with few exceptions, the London Matriculation is the only preliminary examination which will permit a candidate to proceed to the higher examinations in order to become a graduate of the University of London.

Wherever he proposes to study, and whatever qualification he intends to get, the courses of study which a medical student has to undergo are really very much the same; they may be divided, generally speaking, into the two groups of the preliminary and the clinical. But it is not, as a rule, compulsory to undergo the necessary courses of instruction in these two divisions of medical education at the same school. Often a student will be well advised to undergo his preliminary training at a provincial school, or as a student of the University of London, and to pursue his clinical studies elsewhere, as in one of the large Metropolitan hospitals.

The following is a list of the examining bodies, and the degrees and diplomas granted by them which admit the graduate or diplomate to enter his name in the register of legally qualified medical men :—

| Examining Body. | Qualification. |
|---|---|
| **ENGLAND.** | |
| University of Oxford ... | M.B., B.Ch. (Only obtainable after graduating in Arts.) |
| University of Cambridge ... | M.B., B.C. |
| University of London ... | M.B., B.S. |
| University of Durham ... | M.B., generally M.B., B.S. |
| University of Birmingham... | M.B., B.Ch. |
| University of Liverpool ... | M.B., Ch.B. |
| Victoria University of Manchester ... ... ... | M.B., Ch.B. |
| University of Leeds ... ... | M.B., Ch.B. |
| University of Sheffield ... | M.B., Ch.B. |
| University of Bristol ... | M.B., Ch.B. |
| Conjoint Examining Board in England ... ... ... | M.R.C.S., L.R.C.P. |
| Society of Apothecaries of London ... ... ... | L.M.S.S.A. |
| **SCOTLAND.** | |
| University of Edinburgh ... | M.B., Ch.B. |
| University of Glasgow ... | M.B., Ch.B. |
| University of Aberdeen ... | M.B., Ch.B. |
| University of St. Andrews... | M.B., Ch.B. |
| Conjoint Examining Board in Scotland ... ... ... | L.R.C.S.Edin., L.R.C.P. Edin., and L.R.F.P.S. Glasg. |
| **IRELAND.** | |
| University of Dublin ... | Lic. Med., Lic. Surg. (This proviso does not apply to Licentiates.) M.B., B.Ch., B.A.O. (Only conferred on graduates in Arts.) |
| National University of Ireland ... ... ... | M.B., B.Ch., B.A.O. |
| Queen's University, Belfast | M.B., B.Ch., B.A.O. |
| Conjoint Examining Board in Ireland ... ... ... | L., L.M.R.C.P.I., L., L.M.R.C.S.I. |
| Apothecaries' Hall of Ireland | L.A.H.Dub. |
| **WALES.** | |
| University of Wales ... | M.B., B.Ch. |

